# Multi-Method, Partner-Engaged Process to Document Adaptations for ATTAIN NAV: Family Navigation for Autism and Mental Health

**DOI:** 10.1007/s10488-025-01452-z

**Published:** 2025-06-09

**Authors:** Isaac Bouchard, Kassandra Martinez, Pollyanna Gomez-Patino, Felice Navarro, Lauren Brookman-Frazee, Kimberly J. Holmquist, Sonya Negriff, Miya Barnett, Sarabeth Broder-Fingert, Nicole A. Stadnick

**Affiliations:** 1 Department of Psychology, California State University Northridge, Northridge, CA USA; 2 Doctoral Program in Clinical Psychology, San Diego State University, University of California San Diego Joint, San Diego, CA USA; 3 Child and Adolescent Services Research Center, San Diego, CA USA; 4 Department of Psychiatry, University of California San Diego, La Jolla, CA USA; 5 University of California San Diego Altman Clinical and Translational Research Institute Dissemination and Implementation Science Center, La Jolla, CA USA; 6 Department of Research and Evaluation, Kaiser Permanente Southern California, Pasadena, CA USA; 7 Kaiser Permanente Bernard J Tyson School of Medicine, Pasadena, CA USA; 8 Department of Pediatrics, University of Massachusetts Chan Medical School, Worcester, MA USA; 9 Department of Counseling, Clinical, and School Psychology, University of California Santa Barbara, Santa Barbara, CA USA

**Keywords:** Adaptations, Family navigation, Hybrid trials, Implementation, Health equity

## Abstract

Autistic youth often experience co-occurring mental health needs, yet they have multi-level barriers to accessing needed care. To address these barriers, the ATTAIN NAV (Access to Tailored Autism Integrated Care through Family Navigation) intervention was co-designed with caregiver and healthcare partners and delivered by lay health navigators to facilitate access to and engagement with mental health services for school-age autistic youth. This manuscript describes the multi-method, partner-engaged, longitudinal adaptation process to (1) identify intervention content and implementation refinements prior to the hybrid trial and (2) track ongoing research, intervention, and implementation adaptations during the trial and their impacts on study outcomes. The adaptation processes used the Framework for Reporting Adaptations and Modifications to Evidence-based Implementation Strategies (Miller et al., 2021) to guide data collection and evaluation approaches. From the qualitative co-design activities with caregivers (*n* = 5), primary care providers (*n* = 6), developmental care clinicians (*n* = 4), and health informatics staff (*n* = 3), several intervention content and implementation adaptations were identified and integrated prior to the trial. From the longitudinal adaptation tracking process during the trial, a total of 19 adaptations were documented throughout the implementation trial. The adaptations were related to maintaining the feasibility and acceptability of the study procedures (32%), increasing family recruitment/engagement (26%), increasing the acceptability of the intervention components (16%), increasing physician recruitment/engagement (11%), expanding mental health resources (5%), complying with partnered healthcare organization policy (5%), and increasing navigator workflow efficiency (5%). Findings offer a structured and replicable approach adoptable by non-traditional mental health intervention and implementation research.

## Introduction

Autistic youth experience high rates of co-occurring mental health needs that are often overlooked or inadequately addressed (Joshi et al., 2010; Brookman-Frazee et al., 2018; Lai et al., 2019; Levy et al., 2010; Simonoff et al., 2008). There are individual, societal, and system–level barriers faced by autistic children to access needed mental health services across the care cascade, leading to a limited number of individuals with autism benefiting from evidence-based interventions as part of their mental healthcare (Wood et al., 2015). A starting barrier is in the timely identification of co-occurring mental health needs for autistic children. This is often explained by diagnostic overshadowing whereby core symptoms of autism may overshadow underlying co-occurring mental health needs like anxiety, attention problems, or depression (Reaven & Wainer, 2015). For those who are identified with mental health needs warranting further assessment and treatment, the next barrier is in timely referral and access to specialty mental health care (Vohra et al., 2014). In a study surveying mental health treatment facilities across the US, fewer than half provided services for youth with autism, while only 13% reported having a clinician who was trained to treat youth with autism (Cantor et al., 2020). Community mental health therapists express difficulty in serving autistic youth due to their limited training in effective strategies for autism and co-occurring mental health needs (Brookman-Frazee et al., 2012). Compounding the access barriers are the limited mental health interventions tested for autistic youth with multiple co-occurring mental health conditions, especially those who have lower cognitive functioning, transition-age youth, and/or are from racial/ethnic minority communities (Dickson et al., 2022). Finally, service systems for autistic youth (e.g., early intervention, school-based, medical, mental health) are notoriously disconnected, resulting in confusion among providers, caregivers, and autistic individuals (Maddox et al., 2021). The fractured service system landscape for autistic youth underscores the need for integrated care models to hasten access to and engagement in needed and high-quality services.

Primary care is a principal point of care and mental health screening has been recommended as the “eighth vital sign” in pediatric well-child visits (Jellinek & Murphy, 2021). Integrated behavioral healthcare models, defined as care arrangements within primary care settings that promote continuity between physical and behavioral health services, can support the mental health needs of autistic youth (Farmer et al., 2014). To capitalize on the reach of primary care and potential for early identification of mental health needs early for autistic children, our team co-designed a tailored mental health integrated care model, “Access To Tailored Autism Integrated Care” (ATTAIN) for school-age children with ASD in pediatric primary care settings (Stadnick et al., 2019). ATTAIN included a mental health screening and augmented referral process for autistic youth in pediatric primary care. ATTAIN aimed to promote engagement with mental health services by first screening patients using the Pediatric Symptom Checklist-17 (PSC-17) (Murphy et al., 2016). If patients had an elevated PSC-17 score, their primary care provider reviewed the results with the family and offered a referral to mental health services. Based on the healthcare organization, a team of care coordinators or customer service specialists facilitated follow-up with families to support mental health service linkage. Based on the ATTAIN pilot study, this model was feasible and acceptable but several areas for refinement were identified by primary care providers and caregivers (Stadnick et al., 2019). These related to reducing burden on primary care providers and staff during the PSC-17 scoring and referral follow-up process and providing more personalized navigation support to families to secure linkage to mental health and other needed services.

Family navigation is a promising and evidence-based care management strategy that may address these implementation and effectiveness gaps (Godoy et al., 2019; Feinberg et al., 2021). Broder-Fingert and colleagues (2020) identified and defined core components of family navigation for autism based on a synthesis of data from four clinical trials of family navigation to accelerate time to an autism diagnosis. A total of 11 core components were identified and categorized into three distinct domains: Training and Supervision, Navigator Activities, and Navigator Tools. The Training and Supervision domain functions to train navigators on effective strategies such as motivational interviewing, while providing check-ins to help navigate challenging cases and maintain fidelity. The Navigator Activities domain acts to facilitate referral processes and access to responsive services. The Navigator Tools domain serves to provide essential information for navigators in increasing access to services by providing a template for family-specific action plans as well as training manuals. Components within the Training and Supervision domain include training, ongoing supervision, and fidelity monitoring. Navigator Activities include referral to navigation, linguistic and cultural brokering, completing encounters (i.e., navigator and family interactions), barrier identification, emotional support, and care coordination. Navigator Tools includes the navigator workbook, which provides a family-specific action plan template, a motivational interviewing training manual, psychoeducational materials, and family resources. The Navigator Checklist provides a checklist of family-specific navigator tasks. ATTAIN NAV built on this foundation by adapting components to promote early access to and engagement with mental health services for autistic youth, incorporating strategies to improve cross-system collaboration and caregiver engagement.

While family navigation for autism has been clearly operationalized and there is mounting evidence for its effectiveness in early identification and intervention for autism (Feinberg et al., 2021; Feinberg et al., 2016), there remains a gap in supporting school-age autistic youth with co-occurring mental health needs. To our knowledge, there are no published studies that have specifically examined family navigation for school-age autistic children with co-occurring mental health needs. In response, our team endeavored to leverage insights from the ATTAIN program (Stadnick et al., 2022) and adapt the core components of family navigation for autism for this older, clinically complex pediatric population in a NIH-funded study (R34MH120190).

Tracking and considering adaptations in implementation research is essential for ensuring intervention uptake, contextual fit, and effectiveness (Damschroder et al., 2009). Adaptations to intervention content and/or delivery may be essential to optimize their appropriateness for diverse populations, narrow health and services disparities, and be responsive to dynamic contexts (Bernal & Domenech Rodríguez, 2012; Chambers et al., 2013). It is equally important to adapt and track implementation strategies to promote a systematic, replicable pathway for implementation in real-world settings and promote optimal implementation outcomes (Proctor et al., 2011).

The Framework for Modification and Adaptations Expanded (FRAME; Wiltsey Stirman et al., 2019) and the Framework for Reporting Adaptations and Modifications to Evidence-based Implementation Strategies (FRAME-IS) are tools to systematically document adaptions to interventions and their implementation strategies (Miller et al., 2021). These frameworks allow researchers to track a variety of adaptation characteristics (e.g., the nature of the adaptation, the primary goal of the adaptation, the timing of the adaptation) for planning, tracking, and evaluation purposes. Emerging literature has been using the FRAME and FRAME-IS to define and evaluate adaptations in implementation research, including a recent qualitative study that identified retrospective adaptations post-implementation of family navigation for autism (Levinson et al., 2023).

The goal of this manuscript is to describe the multi-method, partner-engaged, longitudinal adaptation process to (1) identify proactive intervention content and implementation refinements prior to hybrid effectiveness-implementation trial and (2) track emergent research, intervention, and implementation adaptations made during the trial and their impacts on study outcomes.

## Methods

This manuscript reports findings from a larger hybrid effectiveness-implementation trial of ATTAIN NAV (Access to Tailored Autism Integrated Care through Navigation, NCT05344378) that compared family navigation, with and without technological enhancements, delivered by lay health navigators to caregivers of youth with autism and co-occurring mental health needs. The goal of the ATTAIN NAV trial was to enhance ATTAIN by adding a family navigation component to improve service navigation for families to needed services and address organizational-level implementation barriers (e.g., insufficient provider time to complete screening, referral, and service linkage) to facilitate screening and referral. The current study focuses on the formative, adaptation work prior to the trial and the pragmatic adaptation tracking system to monitor modifications during the trial. The current study has two goals: (1) describe the process of adapting family navigation content and training materials via pre-implementation interviews and (2) report findings from real-time adaptations tracked during the implementation trial. The clinical and implementation outcomes of the ATTAIN NAV trial will be reported in a separate manuscript. We briefly describe the family navigators, their training, and intervention delivery for context. Following, we describe the methods used to adapt the family navigation intervention and the subsequent adaptation tracking method used during the ATTAIN NAV trial. IRB approval was obtained for data collection and analysis (MASKED FOR REVIEW).

### Family Navigator Background and Training

The navigators were hired through an open university job search that required the following hiring criteria: professional experience in health education, service navigation or related field, bilingual in Spanish and English, and preference for caregivers of neurodiverse children. The positions were part-time (25%). There were two original navigators, both women, hired. One original navigator had worked for the County of San Diego for several years as a social worker, was in the process of finishing her Master of Public Health degree, and she was a mother of a child with developmental needs. The second original navigator had worked as a medical interpreter for Latino families for several years. She left the study after six months to pursue a full-time position. The third navigator who replaced the second original navigator held a Master Degree of Public Health, had worked for many years as a Health Educator for a local non-profit community clinic, and was a mother of a child with developmental needs.

Family Navigators were required to complete the following IRB trainings prior to working with the families: Human Subjects Research, Good Clinical Practice- Social and Behavioral Research Best Practices. The Study Coordinator and Principal Investigator met with Family Navigators conducted an 8-hour training during their first month. The interactive training detailed the ATTAIN NAV study design, methods and procedures, the role of the family navigator to help families connect to mental health and other health services or resources, discussion of barriers for accessing mental health services for school-aged children with autism, tools to overcome the barriers while working with families, coordinating services, communication with patient’s care team, working with families to provide support, explaining systems and accessing outside resources was provided to the family navigators. Additionally, examples of utilizing motivational interviewing, problem-solving education, collaborative decision, psycho-education, community resources and care coordination were provided to the family navigators. Throughout the training, comprehension checks were embedded and multiple opportunities for family navigators to reflect on the content and post questions were offered. The family navigators were also trained in team-based care, effective communication and cultural competence to be able to deliver a culturally sensitive and competent family navigation to all the families enrolled in the ATTAIN NAV study.

Upon completion of the 8-hour initial training, the Study Coordinator (a doctoral student in Clinical Psychology) met with the family navigators to practice and role-play conducting family screening and consenting calls. The Principal Investigator and Study Coordinator were available daily to answer any questions that arose during family navigation screening calls and sessions. Bi-weekly clinical supervision was also led by the Principal Investigator (licensed psychologist), Study Coordinator, and a licensed marriage and family therapist at the partnering healthcare organization.

### ATTAIN NAV Intervention and Delivery

Family navigators were tasked to (1) perform behavioral health screenings and referrals, (2) support access to behavioral health services, (3) helping families to engage in evidence-based treatment, (4) weekly progress monitoring to achieve family goals, (5) family strengthening and empowerment to advocate for their child, and (6) connect to appropriate mental health, developmental, and community-based services. Eligibility for families included: (1) had a child ages 4–16 years old with a documented autism diagnosis, (2) was referred by their child’s pediatrician who was employed at a participating clinic or self-referred in response to an email invitation based on their child’s eligibility, (3) their children scored in the clinically elevated range of the Pediatric Symptom Checklist-17 (PSC-17) (Murphy et al., 2016). Family navigators worked with their assigned families for up to four months or until their family’s service navigation goals had been reached.

ATTAIN NAV started with the creation of the Family Development Plan which consisted of developing 1–3 SMART (Specific, Measurable, Achievable, Relevant, and Time-bound) goals to support the child’s behavioral and mental health needs. These goals were collaboratively set and could include service navigation to and engagement with mental health services, autism-specific services, school-based programs, socio-recreational programs, and parent mental health resources. Navigators met approximately weekly with each assigned family for 15 to 60 min via phone, Zoom, or in-person in the family’s home or a community setting. Although family navigation was not a 24 h service, the family navigators were flexible to accommodate family schedules during the work week (Monday-Friday), including evenings. Examples of family navigator activities included three-way calls between the caregiver and mental health service representatives to schedule appointments, guiding caregivers through submitting intake paperwork for developmental disability services, in home support services, and family medical leave requests, and role-playing with caregivers how to advocate for services for their child in upcoming provider meetings.

Upon completion of the SMART goals or after four months, a final session was scheduled with the family. In the final session, the goals of family navigation were summarized along with strengths and barriers that the family overcame during the family navigation process. The family navigator addressed any pending items or questions from the family and the session was finalized by thanking the family for their participation in the study. Trial outcomes will be reported in a forthcoming manuscript.

### Procedures for Adapting the Family Navigation Intervention and Implementation (Current Study)

Caregivers of autistic youth (*n* = 5), primary care providers (*n* = 6), and developmental care clinicians from the partnering healthcare organization (*n* = 4) participated in individual or group interviews to adapt the family navigation intervention content, navigator activities, and navigator training to be compatible with the service and clinical needs of autistic youth ages 4–16 with co-occurring mental health needs. Health informatics staff—that is, individuals familiar with information technology and embedded research processes— (*n* = 3) participated in individual or group interviews to facilitate the pragmatic design of technology implementation tools to support navigation.

In terms of recruitment, caregivers of eligible children (ages 4–16 years with an autism diagnosis and a patient of a participating clinic) were sent an email from the participating healthcare system. Eighteen caregivers responded to the email expressing interest to participate in an interview. We only collected contact information from those who expressed interest so we cannot provide comparisons between those who participated in an interview and those who declined or did not respond to our follow-up outreach. Those who expressed interest were contacted by the study team to screen for eligibility, and provide more details about participation. All caregiver interviewees participated in individual interviews.

Providers were recruited through nominations by key partners at the healthcare organization for their expertise in service coordination and clinical care for autistic children at the participating clinics. Providers participated in one individual interview and three group interviews, based on their availability. The providers included case managers, primary care physicians, and mental health clinical leaders. One of the primary care physicians had participated in the ATTAIN trial. Two of the developmental case manager clinicians had been involved in advisory activities for the ATTAIN trial. The caregivers and system analysts were not involved in the original ATTAIN trial.

Three system analysts were nominated by the PI of the partnering healthcare system for their expertise in electronic health record integration and health informatics; all these individuals agreed to participate in an interview. System analysts participated in separate individual interviews. All providers and system analysts interviewed were employed by the same partner organization.

All participants were compensated $40 for their participation. Caregivers were interviewed by the PI, a licensed clinical psychologist and an advanced clinical psychology doctoral student with formal training in qualitative methods and clinical interviewing. Providers and system analysts were interviewed by the PI, a licensed clinical psychologist. Interviews occurred before the start of the family navigation trial, lasting from 30 to 60 min in length. Interviews were conducted before the trial while adaptations were made during the trial. These included adaptations made to family navigation trainings as well as delivery. Table 1 details the adaptations that were informed by the interviews.

Interview guides were developed based on the core modules and questions of the FRAME-IS. Core modules of the FRAME-IS include identifying: (1) the evidence-based practice, implementation strategy, modification; (2) what is being modified; (3) the nature of the modification; and (4) the goal of the modification. The interviews were intended to be deductive, targeted and semi-structured. Following a brief overview of family navigation, interviewees were asked to share their perceptions of the goals of family navigation for autism and mental health in primary care. Questions included: What initially comes to mind that should be considered when adapting family navigation? What would you want family navigation to help you with the most related to mental health screening, referral, and engagement? Interviewees were then asked about potential context and delivery modifications. Questions included: Which activities need to be modified for families and their children with autism and co-occurring mental health needs? What modifications are needed to the way specific aspects of family navigation are delivered? Health informatics staff were also asked: How can we best support family navigation within [this healthcare] system? All interviews and focus groups were audio-recorded and transcribed. Interviews were transcribed by a graduate research assistant and then reviewed by the PI and two project staff for accuracy. See Appendix A for the caregiver interview guide and Appendix B for the provider interview guide.

### Procedures for Tracking Adaptations and their Impacts on Outcomes (Current Study)

**Fig. 1:**
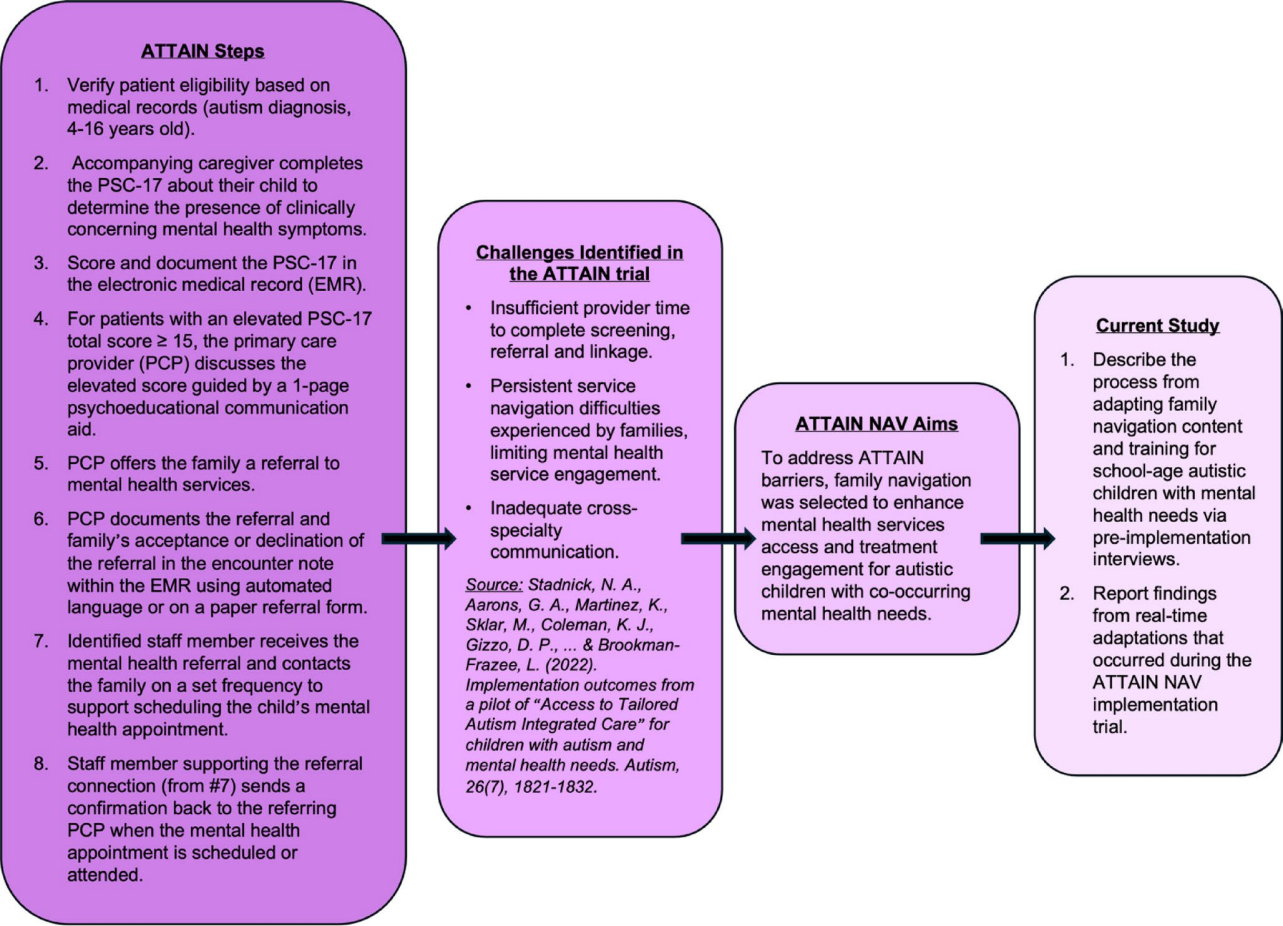
Overview of the ATTAIN trial process, emerging challenges, ATTAIN NAV aims, adaptation objectives, and the focus of the current study

Adaptations were tracked and evaluated during the ATTAIN NAV hybrid trial through a longitudinal, systematic approach. First, an adaptation tracking database was created that included relevant FRAME-IS (Miller et al., 2021) components. The FRAME-IS components that were included in the database focused on characterizing the type, rationale, source, timing, and outcome of the adaptation. Table 1 reports each element in the adaptation tracking database and a corresponding definition. Following the adaptation tracking database development, an adaptations team was assembled that included the PI, family navigators, a research assistant, and the project manager. A member of the adaptations team sent weekly emails to the larger research team and operational partners at the participating healthcare organization to solicit identification of emergent adaptations. The adaptations team also noted any planned or emergent adaptations during their weekly research team meetings, and subsequently documented them in the adaptations tracking database. Figure 1 illustrates the role of pre-implementation interviews in the context of the ATTAIN and ATTAIN NAV studies.

### Data Analysis

For the pre-implementation interviews, rapid qualitative assessment procedures were used to identify themes (Hamilton, 2013). Specifically, the coding team developed a template to summarize topics covered during each focus group under corresponding domain names. Summarized notes were transferred into a matrix and were reviewed individually and collectively by the coding team (an advanced doctoral student with qualitative research experience and two postbaccalaureate research assistants supervised by the PI). Through consensus discussions, crosscutting and unique adaptation themes (Miles & Huberman, 1994) based on interviewee type (caregiver, provider, or system analyst) were culled, and illustrative quotes were identified to exemplify these adaptation themes. Each identified adaptation provided by interviewees was reviewed and discussed with the family navigation investigative team (co-investigators with expertise in family navigation and children’s mental health services) to determine how best to integrate the recommended adaptations into the family navigation curriculum and navigator training manuals, as well as in the technology implementation tools that supported navigation. Qualitative analyses were aided using Microsoft Excel version 16.53.

For the longitudinal adaptation tracking, the adaptations team convened to review the entries for each adaptation to reach a consensus in the specific details of how the adaptation was reported and the impact of the adaptation on the study outcomes or processes. Adaptations were classified as having positive, negative, or neutral impacts. Positive impacts indicated adaptations that successfully met their intended goals, negative impacts indicated adaptations that hindered their intended goals, and neutral impacts had no effect. During meetings, the coding team, comprised of the PI, advanced doctoral student, and a postbaccalaureate research assistant, discussed potential adaptation themes through think-aloud exercises to demonstrate thought processes behind themes (Van Someren et al., 1994). Discrepancies were discussed until an agreement was reached among the coding team. Any disagreement in an adaptation detail was resolved through team discussion. Descriptive statistics and visualizations were used to characterize the documented adaptations and their perceived impact on implementation outcomes. Quantitative analyses and visualizations were conducted in Microsoft Excel version 16.53.

## Results

### Qualitative Themes To Inform Intervention and Implementation Adaptations

#### Modifications To Navigation Goals

Overall, interviewees expressed the strong fit of family navigation for autistic youth with co-occurring mental health conditions. Specifically, interviewees shared that the primary goal of ATTAIN NAV should be to facilitate timely linkage to mental health services, particularly to providers with specialized training in autism and co-occurring mental health conditions. Additional goals for ATAIN NAV were reported by caregivers and providers. These included the importance of providing psychoeducation to caregivers about what to expect from mental health services for their child, the difference between autism-specific versus mental health services, managing wait times for appointments, and how to prepare for an intake assessment.

For example, one caregiver shared, “[The navigator can help with] understanding the next steps [after a mental health need is identified]…if [my child’s pediatrician is] recommending [mental health services] what do I need to do…because like I said, there is just so much stuff.” Another caregiver stated: “In the past, we weren’t given a therapist who worked with autistic children, so a lot of the stuff she was saying was going over his head, so I like this process because then we can get in touch with a therapist that knows how to help us.” One provider shared: “I think [family navigation] sounds perfect…it is often hard for patients with autism, especially as they get older [to navigate the healthcare system…it would be really helpful] from my standpoint, but also from the family’s too.”

Caregivers uniquely expressed that a goal of ATTAIN NAV should include provision of information about ancillary community-based and health services or resources, such as social services, special education services, after school social and recreational programs, as well as mental health sources outside of their healthcare system, if needed. Finally, caregivers reported that ATTAIN NAV should provide resources specific to caregiver wellbeing including support groups, family therapy, mental healthcare for parents.

As one caregiver explained, “I do feel like parents don’t feel they have the support they need or maybe don’t know where to get that information from, so I think [family navigation] would be an excellent resource for everybody. I definitely feel like I do need a little bit of extra help.” Another caregiver shared: “Outside of seeing a therapist and stuff…part of our challenges with navigating is trying to understand what to do with the school for him…that is very challenging…and I remember getting a lot of information but trying to sift through and figure out what makes sense for him [was hard].”

#### Content and Delivery Modifications

Caregivers provided specific guidance on recommended several training topics specific to ATTAIN NAV. These included: an introduction to autism and autism and co-occurring mental health needs, evidence-based practices for autism and co-occurring mental health needs, behavioral health services for autistic youth with co-occurring mental health needs, and an overview of local community health and social service agencies that support autistic youth with co-occurring mental health needs and their families. In addition, caregivers recommended ongoing specialized training for navigators in working with families of autistic youth, such as assessing and addressing caregiver wellbeing and understanding the potential impact of an autism and/or a co-occurring mental health diagnosis on caregiver and family functioning.

As one caregiver reported, “…understanding that this can be challenging…and being aware of where families are in [terms of coping with their child’s diagnosis]…it effects everybody, it’s not just their parents, it’s their siblings too.”

Caregivers also highlighted potential modifications to the delivery of family navigation through ATTAIN NAV to promote accessibility and inclusion. These delivery modifications included expanding navigation sessions to include in-person, telephone, and teleconference options based on the family’s preferences, as well as use of text message reminders and sharing resources through the patient portal.

In reference to family navigation appointment reminders, one caregiver shared, “…to me text messaging is the easiest thing, I don’t really check emails…anything online, any online portal [is helpful].” Another caregiver said: “If it’s just navigation I prefer Zoom, I like the face-to-face more than just being on the telephone and I don’t find it necessary for something like [family navigation] to be in-person.”

#### Modifications To Technology for Navigation Enhancements

Providers shared insights and provided suggestions for engaging providers in the ATTAIN NAV referral process. These suggestions helped guide discussions with health informatics staff as technology enhancements were established.

For example, one provider suggested: “Tell people [pediatricians] how many referrals they sent and tell them what the average is…everybody wants to be above average.” Another provider shared: “What would also help is…when [pediatricians] do send referrals, when [a family navigator sees] the kid, to actually get back to the pediatrician…because that was one of the things with [other service]– we would always make referrals and we would never hear back again, so we had no idea whether [families were connected and engaging with the service], whether it worked, and [that] doesn’t make you excited to make the referral…this way it gives us reinforcement that we did something and it had a positive effect.”

Health informatics staff participants shared several insights into technology enhancements to optimize family navigation. With regards to EMR integration, one informaticist shared, “I think it makes sense to see how we can basically set up a research provider to be able to have those visits/documentation in the Health Connect system and be visible by the patient in their MyChart.”

Informaticists also recommended optimal ways to contact patients, “The problem with [the patient portal] right now is that it is kind of messy with all the COVID alerts so there is kind of an information overload… Text messages were a lot more successful.” As a solution, an informaticist recommended, “Twilio is really easy and inexpensive, like $2 for the number a month and then nominal for each text message.”

With respect to physician dashboards, “We have those tableau dashboards that we like to put in our SharePoint, so within our [MASKED] access-only environment…we could set that up on R&E (Research and Evaluation)…our physicians and providers are very used to this type of access where you give them an internal link and you can just keep it populated however you want.”

### Findings from Tracking Adaptations and their Perceived Impacts on Outcomes

Table 2 displays the 19 adaptations, whether they were planned or unplanned, the rationale/goal of each adaptation, and their link to implementation outcomes. The FRAME-IS informed the data pertaining to the adaptations recorded. The following themes were derived by Module 4, Part 1 of the FRAME-IS which probes the goal of adaptations. A total of 19 adaptations were documented during the implementation trial. Of these, 95% (18) were planned, while 5% (1) were unplanned. Planned adaptations were those that occurred in consultation with the research. Unplanned adaptations were those initiated by those outside of the research team and were implemented without consultation with the research team. The adaptations were related to maintaining the feasibility and acceptability of the study procedures 32% (6), increasing family recruitment/engagement 26% (5), increasing the acceptability of the intervention components 16% (3), increasing physician recruitment/engagement 11% (2), expanding mental health resources 5% (1), increasing navigator workflow efficiency 5% (1), and complying with partnered healthcare organization policy 5% (1). Based on the adaptation team review of perceived impact (see Methods for more details), 68% (13) corresponded with positive implementation outcomes, 21% (4) had no known impact, and 11% (2) corresponded with negative implementation outcomes. Out of the adaptations pertaining to reach (8), 62.5% (5) had positive outcomes, 25% (2) had neutral outcomes, and 12.5% (1) had negative outcomes. Out of the adaptations related to implementation (8), 62.5% (5) had positive outcomes, 25% (2) had neutral outcomes, and 12.5% (1) had a negative outcome. Out of the adaptations pertaining to adoption (2), 50% (1) had positive outcomes while 50% (1) had negative outcomes. All of the adaptations corresponding to effectiveness (2) yielded positive outcomes. Among the adaptation types that corresponded with positive outcomes (13), maintaining the acceptability and feasibility of the intervention components 31% (4), increasing the acceptability of the study procedures 23% (3), and increasing family recruitment/engagement 23% (3) were the most common. Among the adaptation types that corresponded with neutral outcomes (4), increasing family recruitment/engagement 50% (2) and maintaining the acceptability and feasibility of the study 50% (2) yielded neutral outcomes. Among the adaptation types that corresponded with negative outcomes (2), increasing recruitment/engagement of physicians 50% (1) and complying with partnered healthcare organization policy 50% (1) resulted in negative outcomes. The outcomes of the adaptations from the pre-implementation interviews were included in revised intervention materials, family navigator training materials, and implementation process tools (e.g., fidelity tracking forms).

## Discussion

This multi-method study of our partner-engaged adaptation identification, tracking, and evaluation process illustrates the flexible application of the FRAME-IS (Miller et al., 2021) to achieve multiple intervention optimization and evaluation purposes. First, we used the FRAME-IS to build qualitative interview guides that were used with a breadth of key informants to identify priority areas for adaptations needed to the ATTAIN NAV content, navigator training, and study implementation. Second, we used the FRAME-IS to guide operationalization of our longitudinal tracking database that was used through the ATTAIN NAV trial. We note our intentional selection of specific modules of the FRAME-IS to develop each of our data collection tools, and we encourage other FRAME-IS users to consider this approach to maximize the relevance to their project needs.

Our study offers several expansions to the emerging literature on adaptations in implementation research (Rabin et al., 2018; McCreight et al., 2022; McCarthy et al., 2021) and specific to family navigation (Levinson et al., 2023). Adaptations to evidence-based practices may be necessary to promote equitable and effective care delivery across implementation contexts (Barrera et al., 2017; Bernal & Domenech Rodriguez, 2012). Context reigns supreme for implementation as settings have unique needs and dynamic ecological systems that influence intervention-setting fit and may demand adaptations to uphold and sustain an intervention’s benefits and associated implementation strategies. The FRAME-IS facilitated the integration of family navigation delivered by non-traditional mental health providers and tracking adaptations to their delivery of the ATTAIN NAV intervention.

The use of the FRAME-IS in the present study illuminated important adaptations needed for ATTAIN NAV intervention delivery and its implementation strategies related to the infrastructure of the patterning healthcare setting, physicians, and family navigators. Our cross-sectional use of the FRAME-IS for intervention and implementation planning afforded us to gather perspectives from caregivers, clinicians, and health technology experts from the partnering healthcare organization to set up a successful ATTAIN NAV implementation launch. Caregivers provided insight into potential psychoeducation and training that can improve navigation services, such as increased focus on a broad range of health, social, and community services to promote inclusivity and accessibility for caregivers and autistic youth. Clinicians and health technology experts helped identify factors that contribute to feasibility by emphasizing technology enhancements facilitating referral processes and family navigation services. During the longitudinal adaptation tracking phase, the FRAME-IS allowed us to capture a comprehensive catalogue of adaptations to implementation strategies and research activities that could be associated with short-term implementation outcomes. The types of adaptations made to implementation strategies can inform future research on ways navigation services can be improved and how to incorporate nontraditional providers, such as family navigators, in implementation research (Barnett et al., 2021).

Findings from our FRAME-based pre-implementation interviews highlighted areas of family navigation training needed to meet the needs of autistic caregivers and that could be delivered by our family navigators who were non-traditional mental health providers. For example, caregivers emphasized that our family navigators needed to have a strong understanding of the mental health service care cascade from requesting an appointment to being included in decisions about mental health treatment options. Caregivers also recommended that our navigators have training to appropriately address caregiver wellbeing through not only provision of resources that caregivers could access but also through emotional support given the possibility of caregiver strain in caring for a neurodiverse child.

Similar to previous family navigation adaptations research (Levinson et al., 2023), adaptations documented in our project suggested a desire to expand family navigation by facilitating access to families and referring providers and specific recommendations for content and family navigation training topics. Uniquely, adaptations in our project also centered around implementation considerations. This can likely be explained by our use of the FRAME-IS, shifting our lens to the adaptations made to implementation strategies rather than solely the family navigation intervention itself. Adaptations such as the addition of self-referral letters, text message reminders, and incorporating caregiver language preferences promoted engagement for family navigation. These strategies offer guidance for increasing engagement in pediatric primary care trials, specifically for caregivers of youth with autism. Modifications such as onboarding a new family navigator and providing tailored resource guides for caregivers allowed family navigation to be effective despite staffing challenges. By training and leveraging non-specialist providers to deliver family navigation, we offer a feasible approach for integrating non-specialists into pediatric primary care, which may expand workforce capacity and improve engagement with services.

The core components of family navigation for autism served as a strong foundational intervention base for our program. Because our program was intended to meet the needs of older children who have different service needs than infants and toddlers, the focus of the original autism family navigation program, adaptations were necessary not only to the program components but also to the training for family navigators and the implementation process tools (e.g., workflow maps, electronic health record integration) for our implementation context within pediatric primary care. Our study illustrates how adaptation science methods can be used in both a formative way, to adapt intervention and implementation components, and in a longitudinal, documentation manner to characterize the quantity and qualities of adaptation during active implementation. We assert that these adaptation methods are generalizable across settings (e.g., for adapting family navigation and tracking adaptation impact in educational or specialty care settings).

Several limitations are acknowledged. First, we used a purposeful sampling approach to the adaptation selection interviews with caregivers, providers, and health informatics staff, resulting in a small sample size. While small, the perspectives represented in our interviews are from experts with lived experience as a caregiver of an autistic child, as a provider caring for autistic youth, or as a health information systems lead, which provided specific and focused feedback needed for our intervention adaptations. Second, our longitudinal adaptation tracking procedure relied on the self-report of the research team and community partners. Although there were regular outreach efforts to solicit adaptations in real-time, there is a possibility that some adaptations were not reported and thus unable to be tracked. Third, although a call for observed adaptations was solicited on a biweekly basis to reduce retrospective reporting bias, there is still the possibility that not all adaptations were reported or that aspects of adaptations were differently reported. Finally, due to the nature of the funding mechanism, we were not able to track long-term implementation outcomes associated with the adaptations to implementation strategies documented during the ATTAIN NAV trial.

Our adaptation approach offers a replicable method using the FRAME-IS for other implementation and effectiveness research. Adaptations should be expected, they can be planned for, and with systematic tracking and evaluation, they can be valuable to optimize the impact of implementation efforts.

**Table 1:** Adaptation tracking database elements

Database element	Definition
Date of entry/documentation	Date the adaptation was entered
Planned vs. unplanned	Whether an adaptation was planned or unplanned based on the following criteria: - Planned: Adaptations that were discussed and agreed upon with the research team. - Unplanned: Adaptations initiated by others without consultation with the research team
Initiator	The person who initiated or recommended the adaptation
Stage of study	The stage of study when the adaptation was initiated. This included the wedge and clinics involved
Rationale	Why the adaptation was initiated
Goal of adaptation	The purpose of the adaptation
Adaptation in practice	What the adaptation looked like in the implementation setting
Adaptation outcome	The impact of the adaptation rated as positive, neutral, or negative to implementation outcomes (reach, adoption, effectiveness, implementation, maintenance)

**Table 2:** Adaptation details

Adaptation description	Planned/unplanned	Goal	Impact	Implementation outcome
1. Adopted the term, “neurodivergent” in navigation materials when referencing autistic youth	Planned	Increase acceptability of intervention components	Positive	Reach
2. Added a question about caregiver primary language in the screening script	Planned	Increase acceptability of intervention components	Positive	Reach
3. Developed and sent self-referral invitation letters to eligible families	Planned	Increase family recruitment/engagement	Positive	Reach
4. Created a templated text message introducing the study and family navigator that was sent prior to the screening call	Planned	Increase family recruitment/engagement	Positive	Reach
5. Sent a second self-referral letter to eligible families from the first three clinics	Planned	Increase family recruitment/engagement	Positive	Reach
6. Created laminated 1-page project scripts and eligibility criteria for clinic use during family recruitment	Planned	Increase family recruitment/engagement	Neutral	Reach
7. Provided brief talking points about the project to nursing staff to support family recruitment	Planned	Increase family recruitment/engagement	Neutral	Reach
8. The partnering healthcare organization suspended well-child visits for youth aged 6 + during the winter season to prioritize respiratory illness management	Unplanned	Comply with partnered healthcare organization policy	Negative	Reach, Implementation
9. The partnering healthcare organization individually contacted eligible physicians via team collaboration app and email to support physician recruitment	Planned	Increase physician recruitment/engagement	Positive	Adoption
10. The final clinic requested a pre-recorded presentation instead of a live presentation for physician recruitment	Planned	Increase physician recruitment/engagement	Negative	Adoption
11. Ongoing additions to mental health resource list shared with families	Planned	Expand mental health resources	Positive	Effectiveness
12. Created resource guide(s) tailored for parents	Planned	Increase navigator impact	Positive	Effectiveness
13. Adjusted the study design for Step 1 and Step 2 clinics	Planned	Maintain study feasibility/acceptability	Positive	Implementation
14. Trained and onboarded a new family navigator after an original navigator resigned	Planned	Maintain study feasibility/acceptability	Positive	Implementation
15. Created templated letters requesting school accommodations for families	Planned	Increase acceptability of intervention components	Positive	Implementation
16. Postponed the start of ATTAIN NAV at Clinic #4 by one month to accommodate clinic needs	Planned	Maintain study feasibility/acceptability	Positive	Implementation
17. Non-navigator research staff assisted with recruitment during a family navigator’s 1-month leave	Planned	Maintain study feasibility/acceptability	Positive	Implementation
18. Created planned, tailored messages to be sent to families during family navigator’s 1-month leave	Planned	Maintain study feasibility/acceptability	Neutral	Implementation
19. Replaced a clinic following consolidation of clinic sites at the partnering healthcare organization	Planned	Maintain study feasibility/acceptability	Neutral	Implementation

## Appendix A. Caregiver Interview Guide

### ATTAIN NAV Phase 1 Interview Guide—Caregivers

Introduction: “*Thank you for taking the time to meet with me today. I am representing the Access to Tailored Autism Integrated Care through Navigation (ATTAIN NAV) study. ATTAIN NAV is focused on adapting and implementing family navigation to facilitate engagement in mental health services for children with autism. As one of our first steps*,* we are requesting your expertise as a caregiver of a child with autism to adapt the family navigation intervention and delivery to best fit you and your family’s needs. We are asking you to participate in 1–3 individual or group interviews that will each last ~ 1 hour. We are also speaking with KP pediatric providers and staff. There are no right or wrong answers to these questions. You will receive a $40 gift card after participating in each session. Do you have questions before I turn on the audio recorder and begin?”*

**Warmup**.

1. We would like to get to you know and your family a little bit. Please share your name, personal pronouns, and what challenges, inspires, or motivates you as a caregiver/parent of a child with autism?

**Goals of ATTAIN NAV in Primary Care**

*We’ll start by providing a general overview of family navigation for pediatric mental health care and then we’ll talk about adapting it for the ATTAIN NAV project.*

[PROVIDE OVERVIEW OF FAMILY NAVIGATION AND KP WORKFLOW SLIDE]

2. After hearing that description, what are your initial impressions of family navigation?
3. What would you want family navigation to help you with when it comes to your child’s mental health?
  - Prompt: What are most important for family navigators to lead or provide support with? (e.g., administering mental health screening, providing psychoeducation about mental health and eligibility for mental health services, making the call to request mental health services, emotional support)
  - What additional services would be helpful for family navigators to support connecting you or your child with?

**Content and Delivery Modifications**

*Now*,* we’ll discuss potential changes to the family navigation materials and delivery.*

*[SHOW FAMILY NAVIGATION TOPICS]*

4. What topics need to be modified for families of school-age children with ASD + mental health needs?
  - *Probe*: Which topics?
  - *Probe*: Why is the modification needed?
5. How might technology features like the patient portal, telehealth, or e-communication options be useful for family navigation?
  - *Probe*: Do you use the KP patient portal (kp.org) or other virtual/electronic ways to communicate with providers, schedule, or track appointments?
  - *Probe*: What technology enhancements would help you engage with or benefit from a family navigator? (e.g., having access to information sheets, communicate with the navigator)

**Additional Comments**

6. Would you like to share anything else about family navigation for ASD + related to you or your family?

Closing: “Thank you very much for your time and expertise!”

## Appendix B. Provider Focus Group Guide

### ATTAIN NAV Focus Group Interview Guide 1—Navigators

Welcome: “*Thank you very much for taking the time to meet with me today. I am representing the Access to Tailored Autism Integrated Care through Navigation (ATTAIN NAV) study. ATTAIN NAV is focused on adapting and implementing family navigation to help accelerate and facilitate engagement in mental health services for children with autism who may have co-occurring mental health needs (ASD+). As a first step*,* we request your expertise as a service navigator*,* care coordinator or family support partner to adapt the intervention and identify potential individuals who would be interested in training and delivering family navigation. We ask you to participate in a series of up to 3 focus groups that will each last 90 minutes. We will also be speaking with caregivers of children with autism and primary care providers. There are no right or wrong answers to these questions. To maximize our time together*,* I will share pieces of the family navigation intervention and delivery model so you can provide directed comments. I’ll start with more general discussion about your experiences as a service navigator or family support partner to attend to the mental health needs of children with autism and then I’ll move into more specific discussion about how family navigation could best support the kind of work that you do. This will take approximately 90 minutes and you will receive a $40 gift card after participating in each session. Does anyone have questions before I turn on the audio recorder and begin?”*

**Introduction**

We would like to start by getting to know a little about you and your experiences with providing care to families of children with autism or mental health needs in the community. I invite you to introduce yourself by sharing:

1. *Your name*.
2. *Your personal pronouns*.
3. *Your role in your current organization*.
4. *What motivates*,* inspires or challenges you to work in your current role?*

**Goals of Family Navigation for ASD + in Primary Care**

*We’ll start by providing a general overview of what family navigation for pediatric mental health care is and then we’ll guide you through a series of questions about adapting family navigation for the local community.*

**[Spend 15 min reviewing the Foundations of Family Navigation Slides]**.

2. After hearing that description, what initially comes to mind as important considerations for integrating family navigation into the kind of work that you do with families?
  - Prompt: workflow considerations.
  - Prompt: fit with family preferences, values or needs.
  - Prompt: technology considerations (e.g., documentation, receiving/responding to referrals).
  - Prompt: interacting with other care team members.
3. In your role working with families to support service connection, what would you want family navigation to help you with the most related to mental health screening, referral and engagement?

c. Prompt: Are there specific activities that you would like to prioritize family navigators to lead or provide support when it comes to mental health screening, linkage or care engagement? (e.g., administering mental health screening, providing psychoeducation about mental health and eligibility for mental health services)

**Content and Delivery Modifications**

*Now*,* I would like to turn our attention to potential changes to the core activities and delivery of family navigation.*

4. From your review, which activities need to be modified for your families of school-age children with ASD+?
  - *Probe*: What is the nature of the modification? (e.g., tailoring/tweaking/refining language, changes in packaging or materials, removing/skipping elements, reordering)
  - *Probe*: Why is the modification needed?
5. What modifications might be needed to the way *specific aspects* of family navigation are delivered?
  - *Probe*: What? (e.g., format, setting)
  - *Probe*: For whom? (e.g., navigators, clinic staff, families, mental health providers)
  - *Probe*: Why?

**Additional Comments**.

6. Would you like to share anything else about modifications needed to the goals, content or delivery for family navigation for ASD + in your work?

Closing: “Thank you very much for your time and expertise! We will focus the next session on training, implementation and evaluation of family navigation for ASD+.
